# Photosynthetic Electron Transport System Promotes Synthesis of Au-Nanoparticles

**DOI:** 10.1371/journal.pone.0071123

**Published:** 2013-08-20

**Authors:** Nisha Shabnam, P. Pardha-Saradhi

**Affiliations:** Department of Environmental Studies, University of Delhi, Delhi, India; US Naval Reseach Laboratory, United States of America

## Abstract

In this communication, a novel, green, efficient and economically viable light mediated protocol for generation of Au-nanoparticles using most vital organelle, chloroplasts, of the plant system is portrayed. Thylakoids/chloroplasts isolated from *Potamogeton nodosus* (an aquatic plant) and *Spinacia oleracea* (a terrestrial plant) turned Au^3+^ solutions purple in presence of light of 600 µmol m^−2^ s^−1^ photon flux density (PFD) and the purple coloration intensified with time. UV-Vis spectra of these purple colored solutions showed absorption peak at ∼545 nm which is known to arise due to surface plasmon oscillations specific to Au-nanoparticles. However, thylakoids/chloroplasts did not alter color of Au^3+^ solutions in dark. These results clearly demonstrated that photosynthetic electron transport can reduce Au^3+^ to Au^0^ which nucleate to form Au-nanoparticles in presence of light. Transmission electron microscopic studies revealed that Au-nanoparticles generated by light driven photosynthetic electron transport system of thylakoids/chloroplasts were in range of 5–20 nm. Selected area electron diffraction and powder X-ray diffraction indicated crystalline nature of these nanoparticles. Energy dispersive X-ray confirmed that these nanoparticles were composed of Au. To confirm the potential of light driven photosynthetic electron transport in generation of Au-nanoparticles, thylakoids/chloroplasts were tested for their efficacy to generate Au-nanoparticles in presence of light of PFD ranging from 60 to 600 µmol m^−2^ s^−1^. The capacity of thylakoids/chloroplasts to generate Au-nanoparticles increased remarkably with increase in PFD, which further clearly demonstrated potential of light driven photosynthetic electron transport in reduction of Au^3+^ to Au^0^ to form nanoparticles. The light driven donation of electrons to metal ions by thylakoids/chloroplasts can be exploited for large scale production of nanoparticles.

## Introduction

Nanoscience has received immense attention of researchers across the globe owing to its widespread applications for human welfare. The major focus in this area has been (i) to scrutinize mode and mechanisms of synthesis of nanoparticles through physical, chemical and biological means; and (ii) to explore their apt applications in medicine, engineering (including aeronautics and space research), agriculture, cosmetics, therapeutics, food etc. [1]–[3]. In light of advantages of biological means for production of nanoparticles, there has been a rapid increase in identifying the biological factors that are involved in generation of nanoparticles. So far, various biomolecules such as sugars, amino acids, organic acids, phenols [4]–[9] etc. have been shown to have potential to generate metal nanoparticles. Synthesis of nanoparticles has been demonstrated by various research teams using living organisms including bacteria [10], [11], fungi [12] and plants [13].

Potential of photosynthetic organisms, in particular, cyanobacteria (viz. *Anabaena*, *Calothrix*) green algae (viz. *Klebsormidium flaccidum*) [14]–[20] and photosynthetic bacteria (viz. *Rhodopseudomonas capsulata*) [21] in synthesis of Au-nanoparticles has received attention. In cyanobacteria and green algae, Au-nanoparticles were seen in association with thylakoids. Similarly, Beattie and Haverkamp [22] noted the presence of large concentration of Au and Ag nanoparticles in chloroplasts of *Brassica juncea* exposed to respective salt solutions. These investigators believed that chloroplasts are sites for formation of Au-nanoparticles as they contained large concentrations of reducing sugars, glucose and fructose, which are responsible for reduction of Au^3+^ to Au-nanoparticles. Zhang and co-workers [23] reported synthesis of Au-nanoparticles with isolated chloroplasts by vigorously stirring them in incubation medium containing auric chloride in water bath at 25°C for 24–36 h. These researchers claimed that isolated chloroplasts could synthesize Au-nanoparticles with the help of proteins associated with them.

Photosynthetic machinery of thylakoid membranes is a powerful system that draws electrons from water and transport them effectively to terminal acceptor NADP^+^ [24], [25]. It is also well established that this powerful photosynthetic electron transport system can also reduce several other entities such as nitrate, nitrite [26], sulfate [27] and oxygen [28] by effectively donating electrons to them. Inspite of being a powerful system to aptly donate electrons, to the best of our knowledge, no reports of photosynthetic electron transport system of chloroplast to reduce metal ions (such as Au^3+^) and generate nanoparticles are available. It is a well established fact that generation of Au-nanoparticles involves (i) reduction of Au^3+^ to Au^0^ and (ii) nucleation of Au^0^ [29]. This prompted us to evaluate if photosynthetic electron transport system in isolated chloroplasts can donate electrons to Au^3+^ for its reduction to Au^0^ to form nanoparticles. The present investigations were carried out with an aim to validate the role of photosynthetic electron transport system driven by photons in generation of Au-nanoparticles using isolated thylakoids/chloroplasts from leaves of two distinct plant species namely *Potamogeton nodosus* (an aquatic macrophyte) and *Spinacia oleracea* (a terrestrial plant). In this communication, we are reporting for the first time that phototosynthetic electron transport system possess potential to generate Au-nanoparticles.

## Materials and Methods

Fresh leaves of *Potamogeton nodosus* (long leaf pondweed, family Potamogetonaceae, an aquatic macrophyte) and *Spinacia oleracea* (Spinach, family Chenopodiaceae, a terrestrial plant) were chopped and incubated in isolation buffer containing phosphate buffer (400 mM), pH 7.6, 5 mM NaCl, 1 mM MgCl_2_ and 2 mM EDTA for 45 min in dark at 4°C. Leaves were then homogenised in chilled isolation buffer in dark. Homogenate was filtered through 4 layers of Mira cloth and centrifuged at 5000×g at 4°C. The pellet was suspended in suspension buffer consisting of phosphate buffer (200 mM), pH 7.6 and again centrifuged. The pellet was washed 4–5 times with suspension buffer, through suspension and centrifugation, to eliminate the biomolecules (such as phenolics, sugars, free amino acids, free proteins etc., which otherwise can interfere in the process of generation of nanoparticles). Chlorophyll content was determined as per Arnon [30]. In order to check if isolated thylakoids/chloroplasts contained any reducing sugars, their levels were determined following the protocol of Sumner [31].

4 ml of the reaction mixture consisting of different concentrations of Au^3+^, viz. 0, 0.5, 1, 2 mM in phosphate buffer (200 mM, pH 7.6) and thylakoids/chloroplasts equivalent to ∼180 µg chl were exposed to light of ∼600 µmol m^−2^ s^−1^ photon flux density for different time intervals at 24±2°C. Similar set of reaction mixtures consisting of thylakoids/chloroplasts with different levels of Au^3+^ were incubated under similar conditions in dark.

For studying impact of varying light intensities on generation of Au-nanoparticles by thylakoids/chloroplasts, 4 ml of reaction mixture consisting of 2 mM Au^3+^ in phosphate buffer (200 mM, pH 7.6) and thylakoids/chloroplasts of *S. oleracea* equivalent to ∼180 µg chl was exposed to light of varying photon flux density, viz. 0, 60, 300, 600 µmol m^−2^ s^−1^, for different time intervals at 24±2°C.

In order to establish the role of photosynthetic electron transport in reducing Au^3+^ to Au^0^ by donating electrons, two electron transport inhibitors namely DCMU {3-(3,4-dichlorophenyl)-1,1-dimethylurea} and hydroxylamine were tested in accordance with Vani et al. [32].

UV-Vis spectra of reaction mixtures were recorded from 190 to 1100 nm using Specord 200 Analytikjena UV-Vis spectrophotometer. For transmission electron microscopic (TEM) studies, the resultant colloidal solutions were centrifuged at 5000×g for 5 min to sediment down the thylakoids/chloroplasts and 10 µl of the supernatant was drop-coated on 200 mesh copper grid with an ultrathin continuous carbon film and allowed to dry in a desiccator at room temperature. Grids were viewed in the transmission electron microscope (Technai G2 T30) at a voltage of 300 kV. The hardware associated with the machine also allowed (i) energy dispersive X-ray (EDX) analysis to measure the elemental composition; and (ii) selected area electron diffraction (SAED) analysis to determine crystalline nature of nanoparticles.

For powder X-ray diffraction (PXRD) studies, colloidal solutions were centrifuged at 5000×g for 5 min to allow thylakoids/chloroplasts to sediment down and the supernatant was centrifuged at 16000×g to obtain the pellet. The resultant pellet was suspended in distilled water, drop coated on silica surface, dried in desiccator and used for collecting PXRD pattern using Rigaku Rotaflex RAD-B with copper target CuK(α)1 radiation with tube voltage 40 kV and 60 mA in 2 theta (θ) range of 30–80°.

All experiments were carried out independently at least six times and the data was subjected to Duncan's multiple range test to check the level of significance [33].

## Results and Discussion

**Figure 1:**
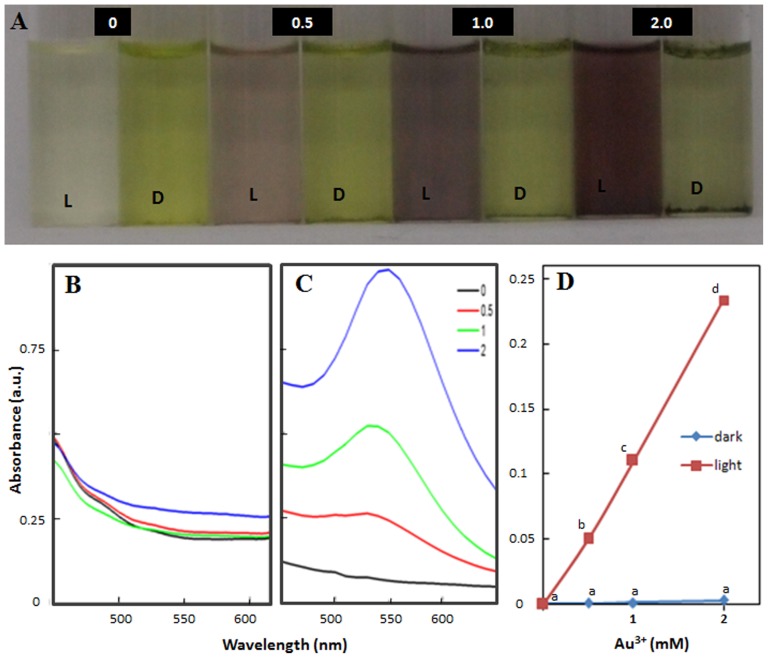
Potential of isolated thylakoids/chloroplasts of *Potamogeton nodosus* to generate Au-nanoparticles in presence of light and dark when suspended in Au^3+^solutions (0, 0.5, 1 and 2 mM). (A) Color of Au^3+^ solutions in dark (D) and light (L); (B) and (C) absorption spectra; (D) absorbance at 545 nm of Au^3+^ solutions incubated in dark and light, respectively. Values represent mean of data collected from six independent experiments. Values designated by different small letters are significantly different at P≤0.05 (Duncan's multiple range test).

Reaction mixtures containing Au^3+^ in which thylakoids/chloroplasts of *P. nodosus* were suspended and incubated in light, turned light pink to purple, depending on the concentration of Au^3+^. The intensity of purple coloration increased with increase in concentration of Au ^3+^ as well as duration of incubation (Figure 1A). Such an alteration in color of Au^3+^ solution to purple is known to be due to generation of Au-nanoparticles [29]. In contrast, no significant alteration in color was noted in Au^3+^ containing reaction mixtures in which thylakoids/chloroplasts of *P. nodosus* were suspended and incubated in dark (Figure 1A). UV-Vis spectra of pink-purple colored mixtures developed by incubating Au^3+^ with thylakoids/chloroplasts in light showed a distinct absorption peak at ∼545 nm (Figure 1C). It is well documented that solutions containing Au-nanoparticles show characteristic absorption peak in range of 500–580 nm that arise due to surface plasmon oscillations specific to Au-nanoparticles [29], [34]–[36]. The presence of peak at ∼545 nm in absorption spectra of pink-purple solutions indicated the formation of Au-nanoparticles from Au^3+^ by isolated thylakoids/chloroplasts in presence of light. Intensity of Au-nanoparticle specific absorption peak increased with increase in (i) concentration of Au^3+^ (Figure 1D) and (ii) duration of incubation of Au^3+^ with thylakoids/chloroplasts in presence of light. As anticipated, no such Au-nanoparticle specific absorption peak was recorded in Au^3+^ solutions incubated with thylakoids/chloroplasts of *P. nodosus* in dark (Figure 1B).

**Figure 2:**
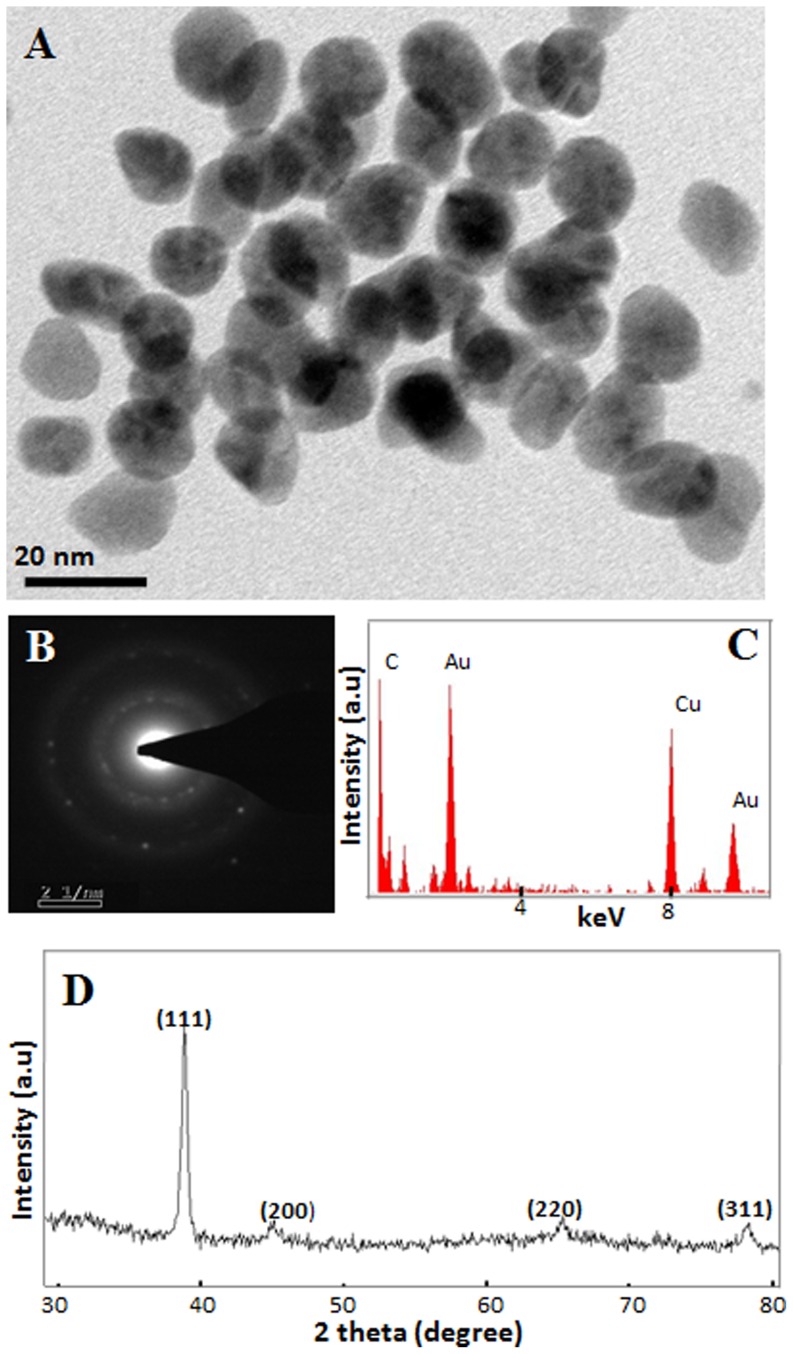
Characterization of Au-nanoparticles synthesized by isolated thylakoids/chloroplasts of *Potamogeton nodosus* in presence of light. (A) TEM image; (B) SAED pattern; (C) EDX spectrum; and (D) PXRD pattern of Au-nanoparticles.

Transmission electron microscopic analysis of the purple colored reaction mixtures showed the presence of Au-nanoparticles which authentically demonstrated that pink to purple coloration of Au^3+^ containing reaction mixtures is indeed due to the presence of Au-nanoporticles. Nanoparticles generated by thylakoids/chloroplasts of *P. nodosus* were in range of 10–20 nm and were mostly spherical (Figure 2A). SAED pattern indicated the crystalline nature of these nanoparticles (Figure 2B) while EDX confirmed that the nanoparticles were composed of Au (Figure 2C). PXRD analysis confirmed the crystalline nature and face centred cubic structure of nanoparticles owing to (111), (200), (220), and (311) Bragg reflections that matched with the JCPDS (Joint Committee on Powder Diffraction Studies) File No. 04-0784 (Figure 2D). These investigations clearly demonstrated that thylakoids/chloroplasts of *P. nodosus* have immense potential to reduce Au^3+^ to Au^0^ and generate nanoparticles in presence of light.

**Figure 3:**
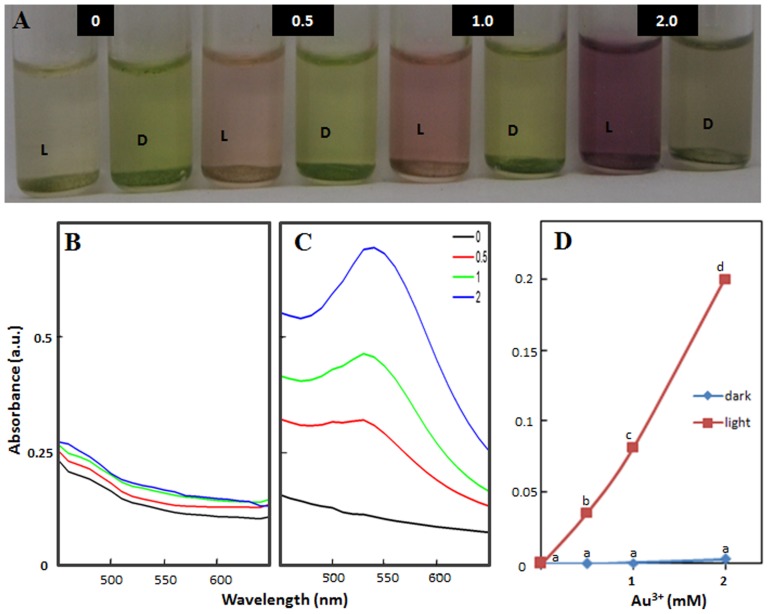
Potential of isolated thylakoids/chloroplasts of *Spinacia oleracea* to generate Au-nanoparticles in presence of light and dark when suspended in Au^3+^ solutions (0, 0.5, 1 and 2 mM). (A) Color of Au^3+^ solutions in dark (D) and light (L); (B) and (C) absorption spectra; (D) absorbance at 545 nm of Au^3+^ solutions incubated in dark and light, respectively. Values represent mean of data collected from six independent experiments. Values designated by different small letters are significantly different at P≤0.05 (Duncan's multiple range test).

**Figure 4:**
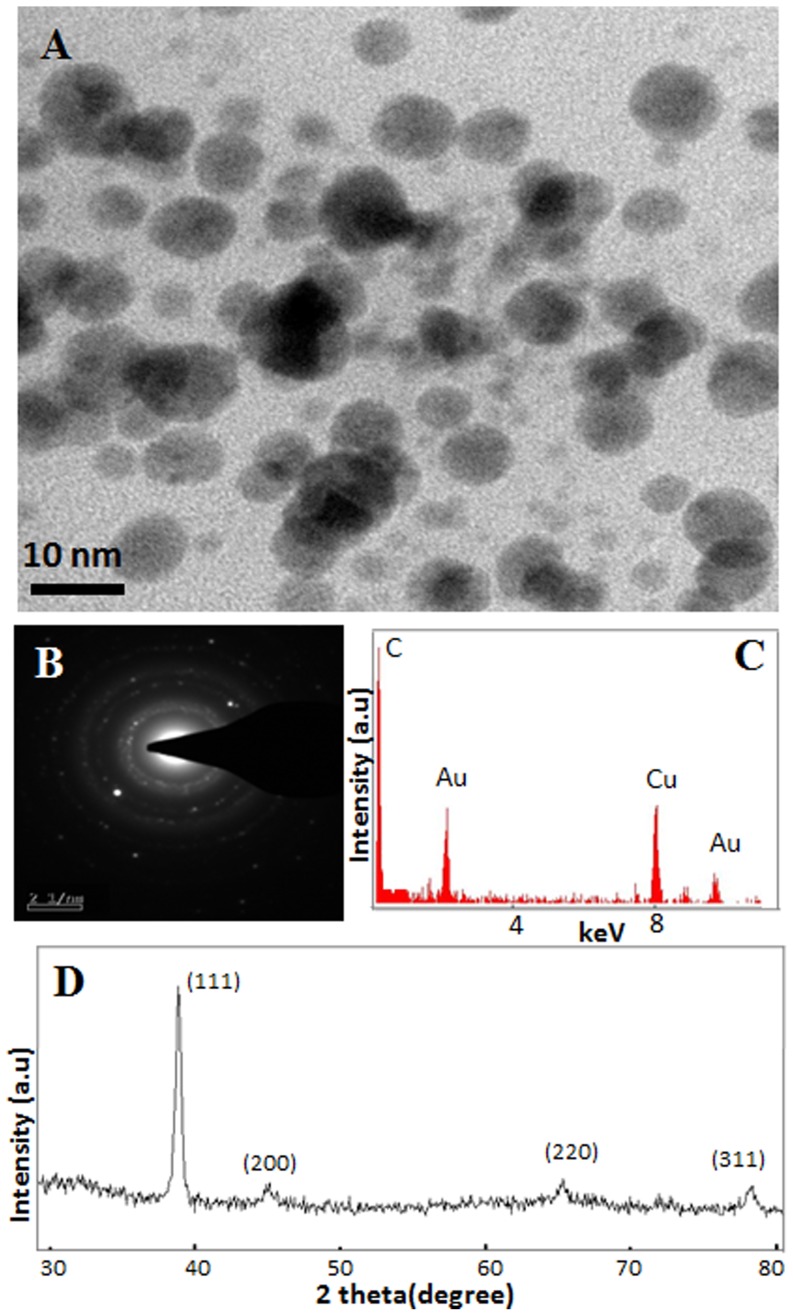
Characterization of Au-nanoparticles synthesized by isolated thylakoids/chloroplasts of *Spinacia oleracea* in presence of light. (A) TEM image; (B) SAED pattern; (C) EDX spectra; and (D) PXRD pattern of Au-nanoparticles.

In order to evaluate if the reduction of Au^3+^ and generation of Au-nanoparticles in presence of light is unique to isolated thylakoids/chloroplasts of *P. nodosus* (an aquatic macrophyte) or is common to thylakoids/chloroplasts from other plant systems, thylakoids/chloroplasts isolated from *S. oleracea* (one of the most widely used plant species for studying various aspects of photosynthetic electron transport in isolated chloroplast) were tested for their efficacy to interact with Au^3+^ solutions and alter their color. Thylakoids/chloroplasts of *S. oleracea* also altered the color of Au^3+^ solutions to pink-purple in presence of light in a manner similar to that noted with thylakoids/chloroplasts of *P. nodosus* (Figure 3A). Accordingly, Au-nanoparticle specific peak in absorption spectra of these pink-purple colored solutions was recorded at ∼545 nm, similar to that noted with thylakoids/chloroplasts of *P. nodosus* (Figure 3C,D). TEM investigations confirmed the presence of Au-nanoparticles which were mostly spherical and in range of 5–10 nm (Figure 4A). EDX confirmed that nanoparticles were composed of Au (Figure 4C) while SAED pattern indicated the crystalline nature of these nanoparticles (Figure 4C). PXRD analysis confirmed that Au-nanoparticles were crystalline and had fcc structure (Figure 4D). Similar to *P. nodosus*, thylakoids/chloroplasts of *S. oleracea* did not alter color of Au^3+^ solutions in dark and hence no Au-nanoparticle specific peak in absorption spectra was recorded (Figure 3A,B). These results confirmed that thylakoids/chloroplasts of *S. oleracea*, like that of *P. nodosus*, have immense potential to generate Au-nanoparticles in presence of light. Therefore, it can be presumed that isolated thylakoids/chloroplasts of all plant species, irrespective of whether they are aquatic or terrestrial, have potential to generate Au-nanoparticles in presence of light.

To the best of our knowledge, prior to this report, only Zhang and co-workers reported generation of Au-nanoparticles by isolated chloroplasts. However, these researchers demonstrated the generation of Au-nanoparticles by constant stirring of chloroplast containing Au^3+^ solutions in water bath at 25°C for 24–36 h, presumely in dark. These researchers believed that proteins associated with chloroplasts are responsible for generation of Au-nanoparticles. As we did not observe generation of Au-nanoparticles by isolated thylakoids/chloroplasts in dark within 24 h, it is unlikely that any of the constituents of thylakoids/chloroplasts directly interact with Au^3+^ to form Au-nanoparticles in dark under ambient conditions. However, it is likely that proteins and other constituents of chloroplasts might have either got released or activated due to vigorous stirring in water bath, which could have played a role in generation of Au-nanoparticles as reported by Zhang and co-workers.

Beattie and Haverkamp [22] reported that Au-nanoparticles are concentrated in chloroplasts of plant *B. juncea* exposed to Au^3+^. As chloroplasts are sites for synthesis of reducing sugars (glucose and fructose), it was presumed by these researchers that these sugars are responsible for generation of nanoparticles. Reducing sugars are known to generate Au nanoparticles [38]. However, during present investigations, no detectable levels of reducing sugars were recorded in isolated thylakoids/chloroplasts of *P. nodosus* as well as *S. oleracea*. Absence of reducing sugars in isolated thylakoids/chloroplasts could be due to (i) thylakoids/chloroplasts isolated as per the details given in materials and methods contains mostly thylakoids [39]; (ii) the same were washed atleast 4 times before they were used for present investigations. Moreover, isolated thylakoids/chloroplasts of both *P. nodosus* as well as *S. oleracea* failed to generate Au-nanoparticles in dark. However, we agree with Beattie and Haverkamp that reducing sugars do play an important role in generation of Au-nanoparticles and therefore reducing sugars produced in chloroplast could have a role in generation of Au-nanoparticles in chloroplast of live plants (i.e. in vivo). As we could not detect any (i) Au-nanoparticle specific color change or absorption peak in Au^3+^ solutions incubated with isolated thylakoids/chloroplasts in dark; and (ii) measurable levels of reducing sugars in association with isolated thylakoids/chloroplasts, we believe that the mechanism of generation of Au-nanoparticles by isolated thylakoids/chloroplasts could be due to light mediated electron transport.

### Mechanism of generation of Au-nanoparticles by isolated thylakoids/chloroplasts

Although, above investigations demonstrated likely role of photosynthetic electron transport in reduction of Au^3+^ and generation of Au-nanoparticles, it is not clear if photosystem II (PSII) and photosystem I (PSI), independently and/or together, promote generation of Au-nanoparticles. As we have not provided any artificial electron donor, we believe that electrons are drawn by PS II under the guidance of oxygen evolving complex from water, which is an established natural electron donor. In order to evaluate if electrons are drawn from water and donated to Au^3+^ through various components of photosynthetic electron transport system, it was necessary to use artificial electron inhibitors like (i) DCMU, which blocks flow of electrons from Q_A_ to Q_B_ [32] and (ii) hydroxylamine, which inactivates oxygen evolving complex (OEC) [32]. OEC promotes stepwise flow of electrons from water to the photooxidized PSII, with simultaneous release of H^+^ and O_2_ [40]. Artificial electron inhibitors are often used for understanding mechanism(s) associated with reduction of various entities (such as NADP^+^, NO_3_^−^) by photosynthetic electron transport system. Unfortunately, during present investigations, these photosynthetic electron transport inhibitors interacted independently with Au^3+^ and formed Au-nanoparticles. Both these inhibitors contain amine groups and it is well documented that amine groups attached to different molecules/compounds have potential to generate Au nanoparticles [29]. Due to these lacunae, such electron transport inhibitors can not be used for probing into the mechanism(s) associated with potential of photosynthetic electron transport to reduce metal ions like Au^3+^ and generate nanoparticles. Therefore, it is important to identify appropriate means and/or inhibitors that can be used for precisely evaluating the contribution made by various components of photosynthetic electron transport system (which includes PSII, plastoquinone, cytochrome b/f complex, plastocyanin, PSI etc.) to redce Au^3+^ and generate Au-nanoparticles.

**Figure 5:**
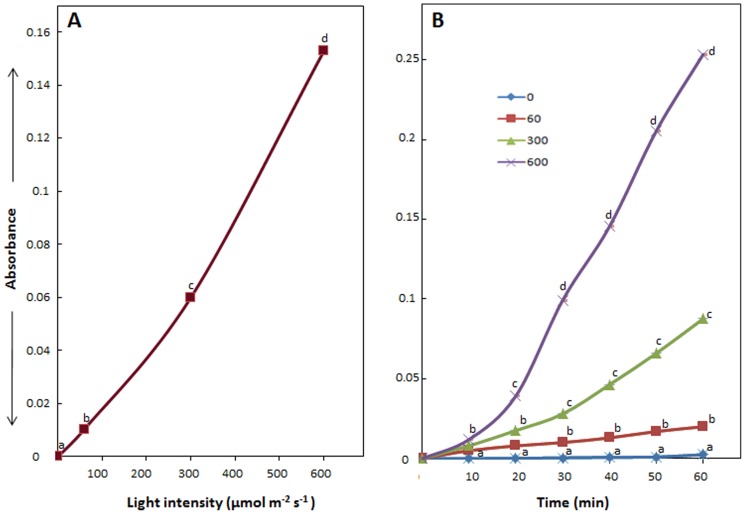
Potential of isolated thylakoids/chloroplasts of *Spinacia oleracea* to generate Au-nanoparticles. (A) Impact of light of varying photon flux density on generation of Au-nanoparticles in 30 min by isolated thylakoids/chloroplasts sincubated in 2 mM Au^3+^; (B) Time dependent variation in generation of Au-nanoparticles by isolated thylakoids/chloroplasts incubated in 2 mM Au^3+^ exposed to light of varying photon flux density (µmol m^−2^ s^−1^). Values represent mean of data collected from six independent experiments. Values designated by different small letters are significantly different at P≤0.05 (Duncan's multiple range test).

It is well documented that photosynthetic electron transport relies significantly on light intensity and in general, increases with increase in PFD. However, in general, photosynthetic electron transport is sensitive to high light intensity [37]. Therefore, to evaluate if the potential of isolated thylakoids/chloroplasts to generate Au-nanoparticles is anyway dependent on light intensity, they were incubated in 2 mM Au^3+^ solution and exposed to light of varying PFD (viz. 60, 300 and 600 µmol m^−2^ s^−1^). As is evident from figure 5A, potential of thylakoids/chloroplasts to generate Au-nanoparticles increased remarkably with increase in PFD. Further, the quantity of nanoparticles generated increased with time irrespective of PFD (Figure 5B). Quantity of Au-nanoparticles generated in 30 min by isolated thylakoids/chloroplasts incubated in 2 mM Au^3+^ solutions and exposed to 300 and 600 µmol m^−2^ s^−1^ PFD was ∼2.4 and 11 folds higher than those exposed to 60 µmol m^−2^ s^−1^ PFD, respectively. The rapid light driven generation of Au-nanoparticles and enhancement in rate of generation of Au-nanoparticles with increase in PFD by isolated thylakoids/chloroplasts clearly pinpoint involvement of photosynthetic electron transport in reduction of Au^3+^, with H_2_O as primary source of electrons.

**Figure 6:**
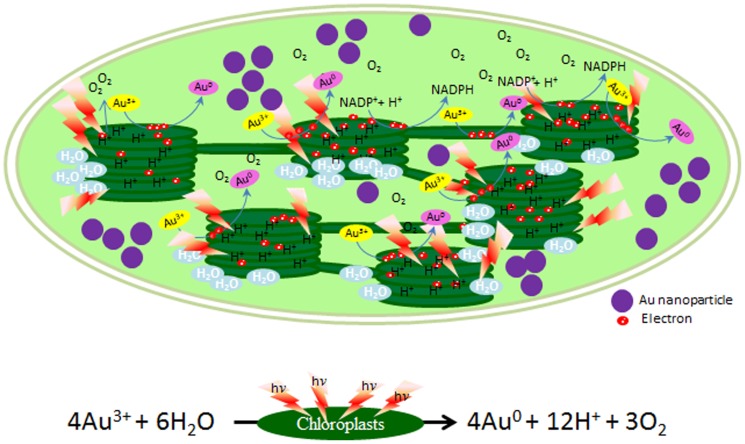
Mechanism for generation of Au-nanoparticles by isolated chloroplasts in presence of light. Photosynthetic machinery driven by light energy splits water into protons, electrons and oxygen. While electrons are transported to NADP^+^, proton gradient is used for generation of ATP. Present investigations support that electrons can also be donated by light energy driven photosynthetic electron transport system to Au^3+^ to form Au^0^, which nucleate to generate Au nanoparticles.

Presence of light harvesting machinery [PSII, PSI and light harvesting chlorophyll protein complex] as an integral part of thylakoid membranes is a well established fact. It is also an established fact that (i) PSII, energized by light, draws electrons from water by splitting it (with simultaneous generation of proton gradient across thylakoids and release of oxygen) and guides their transport to plastoquinone (PQ); (ii) PQ, besides promoting further build up of proton gradient, transports these electrons to cyt b/f complex; (iii) cyt b/f complex transports these electrons through plastocyanin to PSI; and (iv) PSI, energized by light, promotes transport of these electrons to NADP^+^ through ferredoxin [24], [25]. In brief, PSII and PSI of photosynthetic electron transport system, energized by light (photons), drive the transport of electrons from H_2_O to NADP^+^ resulting in formation of NADPH with simultaneous build up of proton gradient (which drives ATP synthesis) and release of oxygen. It is also well established that this photosynthetic electron transport system can also transport electrons from water to other electron accepting entities like nitrate, nitrite, sulfate and oxygen [26]–[28] and bring about their reduction. In a manner similar to the way electrons are transported by photosynthetic electron transport system driven by light from water to various molecules/ions such as nitrate, nitrite, sulfate etc. beside NADP^+^, in all likelihood, the photosynthetic electron transport system must even be guiding donation of electrons to Au^3+^ enabling its reduction to Au^0^ and generation of Au-nanoparticles. Therefore, our findings make us believe that reduction of Au^3+^ by thylakoids/chloroplasts in presence of light is brought about by a simple photochemical reaction (H_2_O→Au^3+^), i.e. electrons are transported from water to Au^3+^, guided by light energized photosynthetic electron transport system as schematically depicted in figure 6.

### Conclusions

The findings presented in this manuscript clearly demonstrated for the first time that isolated thylakoids/chloroplasts, irrespective of whether they belong to aquatic or terrestrial plant system, have immense potential to reduce Au^3+^ to Au^0^ and generate Au-nanoparticles in presence of light and it can be inferred that photosynthetic electron transport system driven by light energy (energy associated with photons) is involved in donating electrons to Au^3+^ (by promoting photochemical reaction H_2_O→Au^3+^). This simple light driven electron transport can be exploited for large scale generation of Au and other metal nanoparticles. To the best of our knowledge this would be the most green, efficient, economically viable and simple natural device for generation of metal nanoparticles.
